# Radio Frequency-Assisted Pasteurization of Cow’s Milk: Process Optimization, Quality Preservation, Shelf-Life Extension, and Economic Assessment

**DOI:** 10.3390/foods15122140

**Published:** 2026-06-13

**Authors:** Sungwan Tuisri, Trisadee Khamlor, Sa-nguansak Thanapornpoonpong, Sukhuntha Osiriphun, Karn Chitsuthipakorn, Vacharapan Trivilatratana, Thanadol Yurak, Watcharapong Naraballobh

**Affiliations:** 1 Department of Animal and Aquatic Sciences, Faculty of Agriculture, Chiang Mai University, Chiang Mai 50200, Thailand; sungvan_t@cmu.ac.th (S.T.); trisadee.kha@cmu.ac.th (T.K.); vacharapan_t@cmu.ac.th (V.T.); thanadol_yurak@cmu.ac.th (T.Y.); 2 Center of Omics for High-Value Agriculture (AgOmics-CMU), Chiang Mai University, Chiang Mai 50200, Thailand; 3 Department of Plant and Soil Sciences, Faculty of Agriculture, Chiang Mai University, Chiang Mai 50200, Thailand; sa-nguansak.t@cmu.ac.th; 4 Department of Food Science and Technology, Faculty of Agro-Industry, Chiang Mai University, Chiang Mai 50100, Thailand; sukhuntha.o@cmu.ac.th; 5 Postharvest Technology Research Center, Faculty of Agriculture, Chiang Mai University, Chiang Mai 50200, Thailand; karn.ck@gmail.com

**Keywords:** radio-frequency, pasteurization, cow’s milk products, economic feasibility

## Abstract

Microbial inactivation is essential for extending the shelf life of raw milk. Radio frequency (RF) thermal pasteurization has emerged as a promising technology for small-scale dairy processing. This study aimed to determine optimal RF temperature–time conditions, evaluate their effects on milk quality across milk from different species of cows, and assess economic feasibility. Raw milk from Holstein Friesian, Jersey, and Brown Swiss cows was treated using a dielectric heating system (40.68 MHz) at 72–92 °C for 20–100 s. The results were compared with conventional low-temperature long-time (LTLT) pasteurization of untreated milk. The optimal condition was 92 °C for 50 s, reducing the aerobic plate count from 5.80 to 0.69 log CFU/mL (a 5.11 log reduction), with no detection of *Staphylococcus aureus*, *Bacillus cereus*, and *Escherichia coli*. RF treatment did not significantly affect milk composition (*p* > 0.05), and color changes remained within acceptable limits. Milk stored at 4 °C maintained quality and safety for up to 28 days. Economic analysis indicated a net present value of USD 134,721.78, a benefit–cost ratio of 3.25, and a payback period of 6.8 months, confirming economic feasibility. These findings demonstrate that RF pasteurization can improve processing efficiency and support sustainable dairy production.

## 1. Introduction

The dairy cattle industry in Thailand has been facing persistent challenges related to production costs. Statistical data from 2020 to 2026 reveal a consistent decline in both the total dairy cattle population and the number of lactating cows. The total dairy cattle population decreased by 30% (from 812,235 to 568,111 heads), while the number of lactating cows declined by 22% (from 307,340 to 239,560 heads). Consequently, the total raw milk production dropped from 1.294 million tons to 1.015 million tons during the same period. Notably, the average productivity per lactating cow remained relatively stable at approximately 4.1–4.2 tons per cow per year, indicating that the reduction in overall milk output was primarily driven by declining cattle numbers rather than reduced individual productivity [1,2]. This situation reflects structural challenges within the dairy sector, affecting consumption levels, food security, and supply chain efficiency from farms and milk collectors to processing plants. Systematic improvements are therefore required to enhance the competitiveness of Thailand’s dairy industry [3]. Northern Thailand is recognized for producing high-quality raw milk under the “Lanna High-Quality Milk” standard, which represents premium milk quality in the region. Favorable climate conditions, environment, and high-quality forage resources contribute to milk that is safe for consumers and rich in protein and fat, comparable to international standards [4]. The primary breeds raised for high-quality production include Holstein Friesian (average yield 26.1 kg/day), Brown Swiss (21.9 kg/day), and Jersey (15.6 kg/day) [5]. Production of premium milk with low somatic cell counts and bacterial loads is essential for market demands, improving product quality, reducing defects, and adding value for both the consumers and producers [6]. Variations in milk fat, minerals, and yield among Holstein Friesian, Jersey, and Brown Swiss cattle depend on both nutrition and genetic background [7]. These compositional differences influence milk quality during pasteurization, as fat, protein, and mineral contents are critical to the functional properties of dairy products after processing [8]. Under identical environmental and feeding conditions, Jersey cows produce milk with higher total solids, which helps preserve milk proteins during processing, and results on higher levels of free fatty acids (FFA). In contrast, Holstein Friesian milk is characterized by a higher mineral content, including calcium, phosphorus, magnesium, zinc, and potassium [9].

Pasteurization is a critical process for extending the shelf life of milk by inactivating pathogenic and spoilage microorganisms [10]. Conventional methods include Low-Temperature and Long-Time (LTLT) pasteurization, in which milk is heated to not less than 63 °C for at least 30 min, and High-Temperature and Short-Time (HTST) pasteurization, in which milk is heated to not less than 72 °C for at least 15 s followed by immediate cooling to 5 °C or lower. Conventional pasteurized milk typically has a refrigerated shelf life of approximately 14–21 days for HTST product, whereas locally produced LTLT milk under prevailing cold-chain conditions in the study region is often limited to about 10 days according to Thai Food and Drug Administration (FDA) [11,12,13]. Moreover, excessive heating temperatures may adversely affect the milk protein quality [14]. Currently, technological advancements have introduced radio frequency (RF) thermal pasteurization as an innovative alternative for milk processing [15,16,17,18]. RF heating utilizes electromagnetic waves in the frequency range of 1–300 MHz [19]. The Federal Communications Commission (FCC) has designated specific frequencies (13.56, 27.12, and 40.68 MHz) to prevent interference with communication equipment [20]. Classified as dielectric heating [21], RF technology has been successfully applied in maintaining paddy rice quality [22], controlling *Aspergillus flavus*, and reducing Aflatoxin B1 contamination [23,24]. RF heating enables rapid volumetric heating, resulting in effective microbial inactivation while minimizing adverse changes in food quality and sensory characteristics [18]. Feasibility assessment is essential to evaluate the applicability of RF technology in the dairy industry. Compared with conventional pasteurization, RF heating has been reported to provide energy savings and lower operational costs [17]. Economic feasibility can be assessed using indicators such as the Net Present Value (NPV), the Benefit–Cost Ratio (BCR), the Internal Rate of Return (IRR), and the Payback Period (PB) [25].

RF thermal processing represents a modern technology for enhancing the value of raw cow’s milk by improving quality, inhibiting microbial growth, and extending shelf life up to three times longer than conventional methods. This advancement provides dairy farmers in Northern Thailand with greater market opportunities due to extended storage duration and enhances the value of premium “Lanna high-quality milk” products. The objectives of this study were: (1) to determine the optimal temperature and time conditions for RF thermal pasteurization of cow’s milk; (2) to evaluate the effectiveness of RF thermal pasteurization under optimal conditions on the compositional quality and shelf life of milk from major dairy breeds in Northern Thailand; and (3) to assess the economic feasibility of RF pasteurization in the dairy production sector.

## 2. Materials and Methods

### 2.1. Materials

A total of 450 L of raw milk was collected from dairy farms in Northern Thailand. The milk was obtained from 10 Holstein Friesian cows, 16 Jersey cows and 10 Brown Swiss cows. All animals were fed a Total Mixed Ration (TMR) containing 12% protein, consisting of 80% roughage (95% corn stover and 5% rice straw) and 20% concentrate (soybean meal, cassava chips, and mineral supplements). Milk samples were freshly collected on the first day of the experiment using automatic milking machines. Raw milk from each breed was pooled separately in cooling tanks and following collection, raw milk was transported under refrigeration and stored at 4 °C, and all pasteurization treatments were performed within 3 h of collection. The samples were then transported to the Laboratory of Milk and Feed Quality Analysis, Faculty of Agriculture, Chiang Mai University, for evaluation of raw milk quality prior to experimentation.

### 2.2. Pasteurization Methods

#### 2.2.1. RF Cow’s Milk Pasteurization System

**Figure 1:**
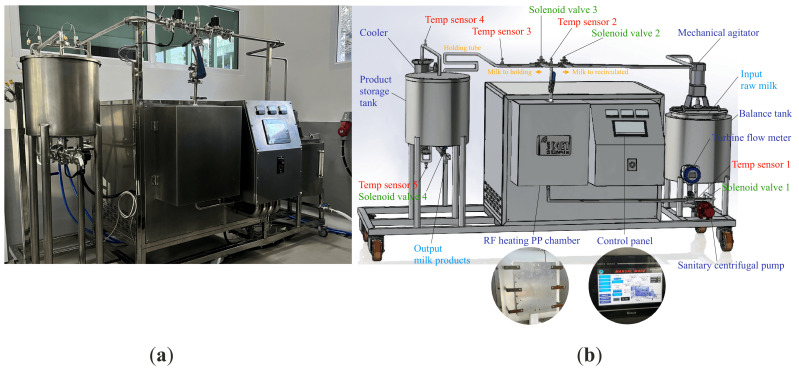
RF thermal pasteurization system: (**a**) prototype unit; (**b**) structural configuration.

Figure 1a illustrates the radio frequency (RF) milk pasteurization system (Application No. 2603000968, Bangkok, Thailand), constructed from stainless steel 304 and designed for automated operation, with processing capacity of approximately 33 L/h. RF heating was applied using dielectric heating at a frequency of 40.68 MHz with a power output of 2 kW (380 VAC). The electrode plates, measuring 25 × 25 cm, were spaced 10 cm apart and positioned around a polypropylene processing chamber connected to the milk flow path, where milk was pumped upward from the bottom Figure 1b. The polypropylene chamber prevented electromagnetic interference. Uniform heating was achieved through impedance matching, optimizing electrical resistance to ensure consistent energy transfer. A VARIAC transformer regulated the voltage, maintaining stable RF heating conditions, with current controlled to not exceed 0.7 A. System operation was managed via a 7-inch PLC touch screen (GL070, Kinco Automation Co., Ltd., Shenzhen, Guangdong, China), allowing precise control of the temperature and milk flow rate. The milk temperature before and after RF heating was monitored in real time using five type-K thermocouples (JBS-3310, SK, Bangkok, Thailand). Prior to RF treatment, milk entered the system through a balance tank, where preheating was performed at 60 °C. This double-boiler tank had a 30 L, internal capacity and was equipped with an adjustable-speed agitator driven by a 0.025 kW, 220 VAC motor (4IK25RGN-C-4GN-25K, Taili Motor Co., Ltd., Taizhou, Zhejiang, China). In the space between the tank walls hot water circulates supplied by a 6 kW, 380 VAC heater (EG-Series, HEAT PLUS, Bangkok, Thailand). The milk flow rate and direction were measured using a turbine flow meter (GTLWGY4BKC2SSNCM4T, GTIMEASURE, Chaohu, Anhui, China), and milk was transferred through the system using a food-grade sanitary centrifugal pump rated at 0.37 kW, 380 VAC (SK-WS-1, FOGO, Wenzhou, Zhejiang, China). If the milk temperature after RF heating did not reach the preset value, the product was automatically recirculated to the balance tank via four electrically actuated stainless-steel valves (VA760, Shinohawa, Taipei, Taiwan; 2 W, 220 VAC). Once the target temperature was achieved, the milk entered a 4.9 m holding tube, ensuring a minimum holding time of 15 s (Table 1). The milk was then cooled via a shell-and-tube heat exchanger connected to a water-cooling chiller (2.4 kW, 220 VAC; K-004.9, DTE, Gaanderen, The Netherlands), reducing the temperature to 4 °C. Finally, pasteurized milk was collected in a sterile 30 L storage tank prior to packaging. When changing the milk source or initiating a new processing cycle, the system was cleaned with hot water at 85 °C for 6 min [15]. After each experimental run, cleaning was performed using a Cleaning-in-Place (CIP) system. The procedure included washing with a 2% sodium hydroxide (NaOH) at 80 °C for 20 min, and was followed by 2% nitric acid treatment (HNO_3_) at 70 °C for 20 min to eliminate pathogens and remove milk residues [26], ensuring post-pasteurization safety [27]. Sanitary verification was conducted using a swab test over a 10 × 10 cm^2^ surface area with a rotating motion. Acceptable microbiological criteria were defined as: Aerobic Plate Count (APC) < 100 CFU/cm^2^, Coliform < 10 CFU/cm^2^, absence of *E. coli*, and no detection of pathogenic microorganisms [28].

The holding time for pasteurization was maintained at not less than 15 s [29], in accordance with safety standards established by the Thai Food and Drug Administration Notifications Nos. 350 (2013), 351 (2013), and 352 (2013) [11,12,13], which are consistent with international guidelines defined by the Codex Alimentarius Commission and the U.S. Food and Drug Administration (FDA) [30,31]. The longer holding times listed in Table 1 correspond to the lowest pump-flow settings used only during preliminary flow-rate screening and were not applied to quality treatments; the conventional LTLT control was held at 63 °C for 1800 s (30 min). Continuous thermal treatment of raw milk was determined by measuring the liquid volume within the holding tube and the flow rate using a turbine flow meter. Pump rotation frequencies were set at 15, 17, and 21 Hz to achieve holding times of 20, 50, and 100 s, respectively. The holding time was calculated as shown in Equation (1).

(1) $$\mathrm{Holding}\;\mathrm{time}=\frac{V_{\mathrm{system}}}{Q}$$

where V system represents the liquid volume within the holding tube which is equals to 0.466 L (based on a tube length of 4.9 m and an internal diameter of 11 mm), and Q denotes the flow rate, expressed in litres per second. To evaluate the pasteurization efficiency of RF thermal processing under different temperature and time conditions, a completely randomized design (CRD) was employed. Each run used 10 L of Holstein Friesian milk, with three replications. Treatments were conducted at temperatures of 72, 82, and 92 °C and holding times of 20, 50, and 100 s (Table 2). Following treatment, the milk was rapidly cooled to 4 °C and aseptically packaged. Results were compared with untreated raw milk and conventional LTLT pasteurization was performed by heating raw milk to 63 °C and holding at this temperature for 30 min (1800 s) in a temperature-controlled, double-jacketed stainless-steel vessel with continuous gentle agitation; the milk was then cooled rapidly to 4 °C and stored refrigerated.

In addition, high-quality cow’s milk produced in northern Thailand was evaluated under the optimal RF processing conditions. This phase included milk from Holstein Friesian, Jersey, and Brown Swiss cows (Table 3). The mixed model designed, was applied to 10 L of milk per breed per run, conducted in triplicate across three experimental cycles under optimal temperature and time conditions. After processing, milk was immediately cooled to 4 °C and aseptically packaged.

#### 2.2.2. Energy Consumption

The specific energy consumption (SEC) of the RF thermal milk pasteurization system [32] was calculated using Equation (2).

(2) $$\mathrm{Specific}\;\mathrm{Energy}\;\mathrm{Consumption}\;(\mathrm{SEC})=\frac{\mathrm{Energy}\;\mathrm{used}}{\mathrm{Product}\;\mathrm{amount}}$$

where SEC represents the specific energy consumption (kWh/L), Energy used refers to the total electrical energy consumed by the RF pasteurization system (kWh), and Product amount denotes the volume of milk processed (L) during continuous pasteurization. Considering the electricity cost in Thailand at 3.98 THB per kWh and using an exchange rate of THB/USD equal to 31.54:1 (as of 2 February 2026), the energy cost per litre (USD/L) was calculated using Equation (3).

(3) $$\mathrm{Specific}\;\mathrm{Energy}\;\mathrm{Cos}t\;(\mathrm{SE}\;\mathrm{Cos}t)=\mathrm{SEC}\times \frac{3.98}{31.54}$$

### 2.3. Analysis of Cow’s Milk Quality

Milk composition (Fat, Protein, Lactose, Total Solid (TS), Solid Not Fat (SNF), Freezing Point (FP)) and Somatic Cell Count (SCC) were analysed using 30 mL samples, with measurements performed in triplicate using a MilkoScan FT2 (FOSS, Hillerød, Denmark) and a Fossomatic FC (FOSS, Hillerød, Denmark), respectively [33,34], both before and after pasteurization. The milk pH was determined using 20 mL samples analysed in triplicate with a bench top pH meter (S-610 Series, PEAK INSTRUMENTS, Shanghai, China). However, somatic cell count (SCC) was determined as a routine raw-milk quality and udder-health grading parameter, to verify that RF processing does not alter the value used for regulatory milk grading; it is not interpreted here as a measure of cell lysis.

### 2.4. Aflatoxin M1 Toxicity Assessment

The aflatoxin M1 (AFM1) concentration was measured using the Reveal Q+ for Aflatoxin M1 test kit, following the manufacturer’s protocol (Neogen Corporation, Lansing, MI, USA) [35]. The assay is based on an immunochromatographic lateral flow competitive method, employing test strips containing antibodies conjugated with colloidal gold particles. When AFM1 is present in milk, the toxin binds to the antibodies, resulting in a reduction in the intensity of the test line proportionally to the toxin concentration. Testing was performed at 65 °C using 0.4 mL of milk per sample. Results were analysed with the Raptor^®^ Integrated Analysis Platform and expressed in parts per trillion (ppt). All measurements were conducted in triplicate, and the values were reported as mean ± standard deviation.

### 2.5. Colour Analysis of the Product

Product colour was evaluated using a spectrophotometer (CM-5, Konica Minolta Sensing, Osaka, Japan) based on light absorption measurements within the CIE Lab* colour system. The Whiteness Index (WI) was calculated according to Equation (4).

(4) $$\mathrm{Whiteness}\;\mathrm{Index}\;\left(\mathrm{WI}\right)=100-\sqrt{{\left(100-L^{*}\right)}^{2}+{a^{*}}^{2}+{b^{*}}^{2}}$$

where L* indicates lightness (higher values = lighter; lower values = darker), +a* indicates redness, −a* greenness, +b* yellowness, and −b* blueness. The total colour difference (ΔE*) was calculated using Equation (5).

(5) $$\mathrm{Total}\;\mathrm{Color}\;\mathrm{Difference}\;\left({\Delta E}^{*}\right)=\sqrt{{\left({\Delta L}^{*}\right)}^{2}{+\left({\Delta a}^{*}\right)}^{2}{+\left({\Delta b}^{*}\right)}^{2}}$$

where ΔL*, Δa*, and Δb* represent the differences between the sample and reference values for each replicate. Measurements were performed in triplicate, and results were expressed as mean ± standard deviation.

### 2.6. Microbiological Enumeration

Microbiological enumeration of RF-pasteurized milk, LTLT-pasteurized milk, and raw milk was conducted in the microbiology laboratory following the AOAC Official Method 986.33 [36]. Samples were serially diluted fivefold by pipetting 1 mL of milk into 9 mL of diluent (diluent 1) and mixing using a vortex mixer. Subsequently, 1 mL of diluent 1 was transferred to diluent 2, and then sequentially from diluent 2 to diluent 3, continuing stepwise until the desired fivefold dilution (or appropriate dilution level) was achieved. All analyses were performed in triplicate. For Aerobic Plate Count (APC), 1 mL of diluted sample was inoculated onto AC Petrifilm (Neogen Corporation, Lansing, MI, USA) and evenly spread over a 20 cm^2^ area. Plates were incubated at 32 ± 1 °C for 48 ± 3 h. Red colonies were counted as positive results. *S. aureus* was enumerated using STX Petrifilm, and *E. coli* using EC Petrifilm (Neogen Corporation, Lansing, MI, USA). In each case, 1 mL of sample was inoculated and evenly distributed over the surface, followed by an incubation at 32 ± 1 °C for 24 ± 3 h. Purple colonies (STX Petrifilm) and red colonies (EC Petrifilm) were recorded as positive results, respectively. Enumeration of *B. cereus* was performed using RAPID’ *B. cereus* Agar Plates. A 1 mL sample was inoculated and spread using a sterile L-shaped spreader to ensure uniform distribution. Plates were allowed to dry for 5 min, inverted, and incubated at 32 ± 1 °C for 24 ± 3 h. Positive colonies appeared red with a surrounding opaque halo. Log reductions were calculated as the difference between the initial microbial count (raw milk) and the microbial count after treatment (log N_0_ − log N).

### 2.7. Shelf-Life Evaluation

A shelf life evaluation was conducted by monitoring microbial counts and pH on days 0, 7, 14, 21, and 28, respectively. All products were stored at 4 °C throughout the study period. Microbiological standards for safety compliance specified that pathogenic microorganisms must meet the following criteria: *Salmonella* spp. must not be detected in 25 mL; *S. aureus* must not exceed 10 CFU/mL (1.00 log CFU/mL); *B. cereus* must not exceed 100 CFU/mL (2.00 log CFU/mL); and *L. monocytogenes* must not be detected in 25 mL. Coliform bacteria are defined as Gram-negative, rod-shaped, non-spore-forming bacteria. *E. coli* must not be detected in 0.1 mL of pasteurized milk. Additionally, Aerobic Plate Count (APC) at the production site must not exceed 10,000 CFU/mL (4.00 log CFU/mL) and must not exceed 50,000 CFU/mL (4.69 log CFU/mL) throughout the distribution period until the labeled expiration date [11,12,13,37,38].

### 2.8. Economic Feasibility Analysis

Economic feasibility was evaluated to determine the potential industrial application of the RF pasteurization technology. Calculations considered electricity costs in Thailand (3.98 THB/kWh), liquefied petroleum gas (LPG) costs (30 THB/Litres), and an exchange rate of THB/USD 31.54:1 (as of 2 February 2026). Four economic indicators were applied [39,40]. The Net Present Value (NPV): the present value of project cash flows discounted at the commercial bank interest rate relative to the initial investment. NPV > 0 indicates economic viability; NPV < 0 indicates non-viability; NPV = 0 indicates neutrality. The NPV was calculated according to Equation (6).

(6) $$\mathrm{NPV}=\sum_{t=1}^{n}\frac{B_{t}}{{\left(1+r\right)}^{t}}-\left(\sum_{t=1}^{n}\frac{C_{t}}{{\left(1\;+\;r\right)}^{t}}+C_{0}\right)$$

where NPV is net present value, B_t_ is project benefits per year t, C_t_ is project costs per year t, r is the discount rate (7.042%), derived from a minimum retail lending rate (MMR) of 6.945% reported by Krung Thai Bank on 22 December 2025, and n is project lifetime (years). Internal Rate of Return (IRR): is the discount rate at which net cash flow equals zero. When IRR exceeds the discount rate, the project is considered economically feasible. The IRR was calculated according to Equation (7).

(7) $$\sum_{t=1}^{n}\frac{B_{t}}{\left(1+\mathrm{IRR}\right)}-\left(\sum_{t=1}^{n}\frac{C_{t}}{\left(1+\mathrm{IRR}\right)}+C_{0}\right)=0$$

The Benefit-Cost Ratio (BCR): is the ratio of the present value of benefits to the present value of costs. A BCR > 1 indicates profitability; a BCR = 1 indicates break-even; and a BCR < 1 indicates non-viability. Equation (8).

(8) BCR=∑t=1nBt1 + it∑t=1nCt1 + it+C0

The Payback Period (PB): is the time required for cumulative cash flow to recover from the initial investment and is calculated as shown in Equation (9).

(9) $$\mathrm{PB}\;\left(\mathrm{year}\right)=\frac{\mathrm{initial}\;\mathrm{investment}\;(\mathrm{USD})}{\mathrm{annual}\;\mathrm{cash}\;\mathrm{flow}\;(\mathrm{USD}\;\mathrm{per}\;\mathrm{year})}$$

### 2.9. Analysis

The data for determining the optimal processing conditions were analysed using a completely randomized design (CRD) with 3 replications and expressed as mean ± standard deviation (SD). Analysis of the variance (ANOVA) following the Tukey’s post hoc test was applied at a significance level of *p* < 0.05. The data from the breed comparison experiment was analysed using a linear mixed model (LMM) and presented as a least-squares means ± standard error of the mean (SEM). The dairy breed (Breed), pasteurization method (Method) and their interaction (Breed × Method) were treated as fixed effects. Mean differences were compared using Sidak’s post hoc test at *p* < 0.05. All statistical analyses were performed using R software (version 4.5.2; R Core Team, Vienna, Austria) and RStudio (version 2026.1.0.392; Posit Software, Boston, MA, USA).

## 3. Results and Discussion

### 3.1. Characteristics of RF Thermal Pasteurization

#### 3.1.1. Pasteurization Temperature Profile

**Figure 2:**
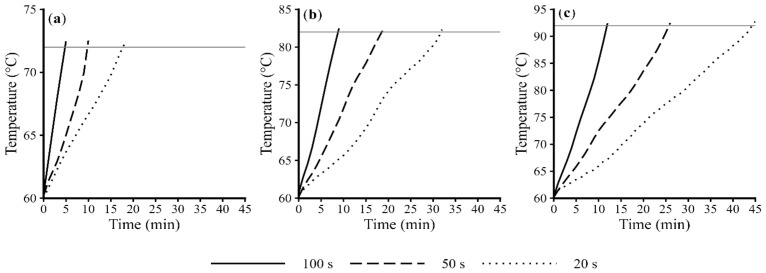
Temperature profiles of milk samples during radio frequency (RF) heating under different holding times at target temperatures of (**a**) 72 °C, (**b**) 82 °C, and (**c**) 92 °C. Solid, long-dashed, and dotted lines represent holding times of 100, 50, and 20 s, respectively. The horizontal grey line in each panel indicates the corresponding target temperature (n = 3).

Figure 2 illustrates the relationship between temperature and time during radio frequency (RF) heating. Temperature increases to 72, 82, and 92 °C were evaluated under holding times of 100, 50, and 20 s, achieved by setting milk flow rates at 77.80, 33.35, and 15.91 L/h, respectively. The results showed that the RF heating duration required to reach the target temperature, as well as the maximum temperature recorded prior to cooling, varied across treatments. At 72 °C (a) (RF72/100, RF72/50, and RF72/20), heating times were 5, 10, and 18 min, with maximum temperatures of 72.46, 72.53, and 72.20 °C, respectively. At 82 °C (b) (RF82/100, RF82/50, and RF82/20), heating times were 9, 19, and 32 min, and the corresponding maximum temperatures were 82.50, 82.50, and 82.33 °C. At 92 °C (c) (RF92/100, RF92/50, and RF92/20), heating times were 12, 26, and 45 min, with maximum temperatures of 92.43, 92.50, and 92.77 °C, respectively.

The temperature of raw milk increased continuously over time before reaching the target temperature. Because RF heating is volumetric and rapid, target temperatures were reached and held within 20–100 s, substantially limiting the cumulative thermal load and the associated risk of whey-protein denaturation relative to prolonged conventional heating; nonetheless, some denaturation at 92 °C cannot be excluded and is discussed below. However, as shown in graphs (a–c), the holding time of 100 s required the shortest time to reach the target temperature, followed by 50 s and 20 s, respectively. The increase in temperature was related to the milk flow rate; a lower flow rate resulted in greater energy absorption, leading to a more rapid temperature rise (∆T). Research on temperature profiles has indicated that the heating efficiency in continuous flow systems may depend on the liquid flow rate and the holding time [41]. In addition, increasing the temperature from 72 °C to 92 °C required a noticeably shorter time. As the milk temperature increased, the dielectric loss factor (ε″) also increased at higher temperatures, resulting in greater energy absorption [42].

#### 3.1.2. Energy Consumption

Table 4 presents the ECC, SEC, and the SE Cost of milk processed using RF thermal pasteurization. The results showed that the ECC increased with both the processing temperature and heating duration, ranging from 7.03 to 9.20 kWh. The RF92/100 exhibited the highest electricity consumption at 9.20 ± 0.2646 kWh, which was significantly higher (*p* ≤ 0.05) than treatments with shorter holding times. In contrast, the lowest electricity consumption was observed at RF72/20, with a value of 7.03 ± 0.1155 kWh. A similar trend was observed for SEC, which increased with holding time. Treatments with a holding time of 100 s showed the highest SEC values, ranging from 0.48 to 0.59 kWh/L, significantly higher (*p* ≤ 0.05) than those at 20 and 50 s. The holding time was therefore identified as the primary factor influencing the increase in energy consumption per unit volume of milk. The SE Cost followed the same pattern as SEC. Treatments with a holding time of 100 s resulted in the highest energy cost, approximately 0.06–0.07 USD/L, whereas treatments with a holding time of 20 s produced the lowest cost, around 0.01 USD/L. No statistically significant differences were observed among temperatures within the same holding time (*p* > 0.05).

The use of higher processing temperatures influences both energy consumption and production cost per unit of product. The temperature and heating duration are critical factors for ensuring food safety. Nevertheless, RF thermal pasteurization provides faster heating than conventional processes [43], thereby reducing problems associated with milk fouling and blockage in the processing pipelines [18]. Compared with small-scale pasteurized milk plants, RF pasteurization demonstrates lower energy consumption per unit of product, highlighting the importance of energy efficiency as a key determinant of the production cost [44]. However, the production cost of pasteurized milk in large-scale factories ranges from 3.76–6.53 USD per ton (approximately 0.0038–0.0065 USD per liter), which is lower; however, farmers in Thailand are unable to own such factories [45].

#### 3.1.3. Effects of Optimal Temperature and Time Conditions on Cow’s Milk Pasteurization

Table 5 presents the chemical composition, physical quality parameters, and somatic cell count of milk pasteurized using RF at 72, 82, and 92 °C for 20, 50, and 100 s. The results indicated that RF pasteurization did not significantly affect major milk components including fat, protein, and TS (*p* > 0.05). The fat content ranged from 2.99 to 3.53%, protein from 3.24 to 3.28%, and TS from 11.88 to 12.23%.

However, the lactose content differed significantly (*p* < 0.05) at temperatures of 82 and 92 °C, particularly at holding times of 50 and 100 s, where values ranged from 4.47 to 4.51% compared with treatments at 72 °C. The SNF ranged from 8.70 to 8.92%, with RF82/100 and RF92/100 showing the highest SNF values, significantly different (*p* < 0.05) and consistent with the observed increase in lactose content.

The SCC ranged from 192.00 × 10^3^ to 215.67 × 10^3^ cells/mL, with no statistically significant differences among treatments (*p* > 0.05), indicating that RF heating does not affect SCC levels in milk. Similarly, the FP ranged from −0.526 to −0.531 °C and showed no significant differences (*p* > 0.05), suggesting that RF processing did not alter the milk water content or concentration after treatment. The milk pH ranged from 6.77 to 6.81 and showed significant differences among treatments (*p* < 0.05); however, values remained within the acceptable quality standards for milk [11].

The RF pasteurization at temperatures of 72–92 °C for 20–100 s show small but statistically significant in lactose, SNF and pH but not effect fat, protein and total solids in milk. However, the reduction of microbial populations in raw milk is essential for food safety. Table 6 presents microbial counts in RF-pasteurized milk. The results indicated that APC decreased as the processing temperature and holding time increased. RF72/20, APC was 2.38 log CFU/mL, whereas RF92/50 and RF92/100 showed a reduction of 0.69 log CFU/mL, representing a statistically significant difference (*p* < 0.05). For the pathogenic bacterium *S. aureus*, detection at 72 °C for 20 s was 1.64 log CFU/mL.

Extending the holding time to 50 s reduced the bacterial load, and no detection was observed at higher temperatures or at 72 °C for 100 s, indicating effective inactivation under these conditions. *B. cereus*, a heat-resistant pathogen, also showed a significant reduction (*p* < 0.05) with increasing temperature and holding time. Counts decreased from 1.34 log CFU/mL at RF72/20 to non-detectable levels at RF92/50 and RF92/100. In addition, coliform bacteria, including *E. coli*, were not detected in any treatment.

Overall, RF heating reduced microbial contamination to levels generally consistent with regulatory standards on day 0. However, the 72 °C/20 s condition showed residual *S. aureus* counts (1.64 log CFU/mL), indicating that this treatment was insufficient for complete inactivation. In contrast, no detectable *S. aureus* was observed at 72 °C/100 s and under all treatments at 82 °C and 92 °C, including the selected 92 °C/50 s condition.

Increasing the temperature and holding time during RF pasteurization had a direct and statistically significant effect on reducing the microbial populations in raw milk, including APC, *S. aureus*, and *B. cereus*. In addition, product colour was evaluated as an important parameter for assessing the impact of RF pasteurization on milk quality. Table 7 presents colour changes in pasteurized milk. The results showed that the lightness value (L*) ranged from 88.26 to 89.85 and decreased as temperature and holding time increased. The highest L* value was observed in RF72/20 (89.85 ± 0.52), which was significantly different (*p* < 0.05) from RF82/50, where the lowest value was recorded (88.26 ± 0.78). The red–green coordinate (a*) was highest at 72 °C and differed significantly (*p* < 0.05) from values at 82 and 92 °C.

As temperature increased, a* decreased toward zero or negative values, indicating reduced redness. The yellow–blue coordinate (b*) ranged from 14.46 to 15.64, with RF92/50 showing the highest value (15.64 ± 0.15), suggesting a slight increase in yellowness at higher temperatures. The Whiteness Index (WI) ranged from 80.48 to 82.32 and decreased with increasing temperature, consistent with the reduction in L* value observed at 92 °C. Nevertheless, WI values remained within the normal range for pasteurized milk products. Total colour difference value (ΔE*) ranged from 1.01 to 2.84, with the lowest value observed in RF72/20 and the highest in RF92/50. Although ΔE* increased with temperature, all values remained below 3, indicating that colour differences were minimal and not perceptible in the final product.

The evaluation of the optimal temperature and time conditions for cow’s milk pasteurization demonstrated that RF processing at RF92/50 was the most suitable condition when considering both product safety and quality. Under this condition, the APC was reduced by 0.69 log CFU/mL, while *S. aureus*, *B. cereus*, and *E. coli* were not detected, indicating a statistically significant microbial inactivation effect. Moreover, the chemical composition of milk including fat, protein, lactose, TS, and SNF showed no significant changes. Colour parameters (L*, a*, b*), the WI, and ΔE* remained within ranges that did not adversely affect product quality. In terms of energy performance, RF92/50 exhibited moderate specific energy consumption and energy costs, reflecting an effective balance between microbial reduction, product quality preservation, and energy efficiency. Comparisons among raw milk (RAW), conventional LTLT pasteurization (63 °C, 30 min), and RF92/50 (Table 8) revealed that fat, protein, lactose, TS, and SNF in RF92/50 were closer to those of raw milk than those obtained with LTLT. In contrast, LTLT showed significantly higher protein, lactose, and SNF values compared with raw milk (*p* < 0.05). The freezing point of LTLT increased to −0.566 °C, suggesting that prolonged heating affected solute equilibrium in milk. The milk pH and SCC did not differ significantly among treatments (*p* > 0.05).

Regarding the microbiological quality, raw milk exhibited a high APC of 5.80 log CFU/mL, with detection of *S. aureus*, *B. cereus*, and *E. coli*. Both LTLT and RF92/50 significantly reduced the APC to approximately 0.68 and 0.69 log CFU/mL, respectively (*p* < 0.05), and neither method detected *S. aureus* or *E. coli*. However, *B. cereus* remained detectable after LTLT at 0.68 log CFU/mL, whereas RF92/50 showed no detection, indicating superior control of heat-resistant microorganisms by RF processing. In terms of colour characteristics, raw milk exhibited the highest L* value (90.53), which decreased with thermal processing. RF92/50 showed the lowest L* value (88.32), with a statistically significant difference (*p* < 0.05). A similar decreasing trend was observed for a*, while b* was the highest in RF92/50, indicating a slight shift toward yellowness. The Whiteness Index decreased with increasing heat intensity, and ΔE* for RF92/50 was 2.84 statistically different but still below the threshold considered perceptible according to established standards (ΔE* < 1 are generally imperceptible and 1–3 perceptible only to trained observers) [46]. Suggesting limited perceptibility to consumers; given that the optimal condition approached ΔE* = 3, consumer sensory confirmation is recommended and is planned in future work.

The condition of RF92/50 was selected as optimal because it produced the greatest microbial reduction (5.11 log reduction in APC, with no detectable *S. aureus*, *B. cereus* or *E. coli*) while preserving milk composition and limiting colour change (ΔE* < 3), at acceptable energy cost. Because the LTLT control (63 °C, 30 min) is not thermally equivalent to the RF treatment, the present design cannot separate temperature-driven from RF-specific (non-thermal) contributions. A conventional treatment matched to the same temperature–time profile is required to attribute the observed effects definitively, and this is identified as priority future work.

Table 9 presents the AFM1 levels in raw milk, conventional LTLT pasteurization, and RF pasteurization. The results showed that raw milk exhibited the highest AFM1 concentration, with a maximum value of 16.20 ng/kg and an average of 13.18 ± 2.11 ng/kg, exceeding those observed after all heat treatments. Following pasteurization by LTLT and RF, maximum concentrations ranged from 8.70 to 11.20 ng/kg, with mean values between 7.23 ± 2.23 ng/kg to 9.83 ± 0.29 ng/kg. Importantly, all mean values remained below the safety limit of 50 ng/kg established by international standards [47].

Statistical analysis indicated that AFM1 levels in raw milk were not significantly different from several pasteurized treatments presented in Table 9, reflecting the high thermal stability of AFM1 and the fact that heating below 100 °C is insufficient for its complete degradation [48]. The observed reduction in AFM1 following pasteurization is likely associated with toxin binding to milk proteins, which may reduce its detectable concentration [49].

### 3.2. Effects of RF Pasteurization on Milk from Different Dairy Breeds Under Optimal Conditions

#### 3.2.1. Chemical Composition, Physical Quality, and Somatic Cell Count

Table 10 presents the chemical composition, physical quality parameters, and SCC of milk from different dairy breeds. Major chemical components-including fat, protein, lactose, TS, and SNF—were significantly influenced by the dairy breed, the pasteurization method, and their interaction (*p* < 0.05). Raw milk generally exhibited higher fat and TS than pasteurized milk. RF pasteurization was associated with slight reductions in fat, protein, and SNF. The lactose content in LTLT-treated milk was significantly higher than in RF-treated milk for Holstein and Jersey breeds, whereas Brown Swiss milk showed no significant difference between raw and LTLT treatments.

The SCC did not differ among breeds, likely reflecting uniform herd management conditions. Pasteurization reduced SCC levels across treatments. The FP varied significantly according to breed and pasteurization method (*p* < 0.05), although no interaction between these factors was observed. Initial pH values differed among breeds and showed a significant interaction between breed and pasteurization method (*p* < 0.001). Nevertheless, the pasteurization itself did not substantially alter the milk pH. Thermal treatment at higher pasteurization temperatures can directly affect milk composition; for example, whey proteins begin to denature at temperatures above 70 °C [50]. While elevated temperatures improve food safety through microbial reduction, they may also induce physical and chemical changes in milk [51].

#### 3.2.2. Product Colour

Table 11 presents the colour characteristics of milk products, including L*, a*, b*, WI and ΔE*. The results indicated that the dairy breed significantly influenced colour parameters (L*, a*, b*) and the WI (*p* < 0.001). Following RF pasteurization, lightness increased across all breeds compared with raw milk and LTLT-treated milk. This change is attributed to structural modifications of casein micelles and fat dispersion, which enhance light scattering within the milk matrix [52]. In addition, exposure of raw milk to higher thermal intensity significantly reduced a* values (*p* < 0.001) across all breeds, indicating a shift toward greener tones. Meanwhile, b* values increased with higher pasteurization temperatures, resulting in a slight increase in yellowness. This effect is associated with whey protein alterations and Maillard reactions involving reducing sugars [53]. Pasteurization did not significantly affect the Whiteness Index, and no interaction between the dairy breed and pasteurization method was observed for colour changes (*p* > 0.05). The consistent direction of change across breeds indicates that the RF effect on colour is largely independent of breed. Among breeds, Holstein milk exhibited the highest WI, followed by Brown Swiss and Jersey. These differences are likely related to variations in milk composition, particularly fat content and casein micelle structure, which influence light scattering properties [54]. The ΔE* increased significantly after RF pasteurization, followed by LTLT and raw milk, respectively; however, all values remained within acceptable limits and below the threshold defined by quality standards [46].

#### 3.2.3. Microbial Counts and pH During Product Shelf Life

**Figure 3:**
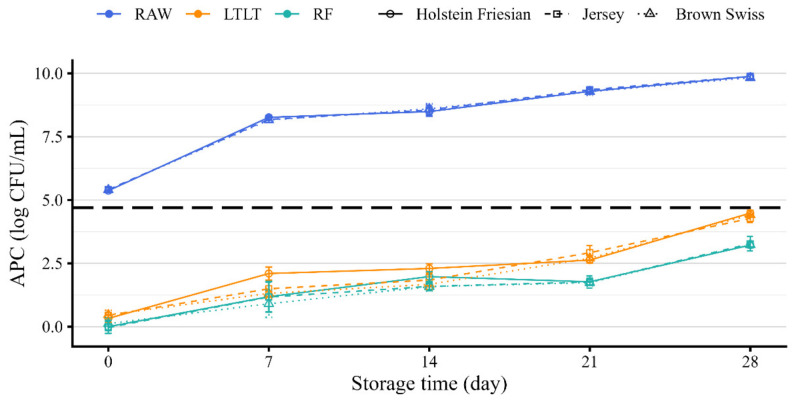
Changes in aerobic plate count (APC) in milk from Holstein Friesian, Jersey, and Brown Swiss subjected to different pasteurization methods (Raw, LTLT, and RF) during refrigerated storage (4 °C) for 28 days. The dashed horizontal line denotes the regulatory APC limit (4.69 log CFU/mL).

Product shelf life was determined based on microbiological safety standards and pH stability during storage at 4 °C [37]. The results of APC analysis are presented in Figure 3. On day 0, the average APC values across all breeds were 5.39, 0.41, and 0.03 log CFU/mL for raw milk, LTLT, and RF treatments, respectively. By day 28, the APC in LTLT-treated milk increased to 4.39 log CFU/mL, approaching the reference limit of 4.69 log CFU/mL. In contrast, RF-pasteurized milk showed a lower increase, reaching 3.24 log CFU/mL. Microbial counts increased progressively during storage due to the growth of psychrotrophic bacteria capable of proliferating at temperatures below 7 °C. Raw milk exhibited the highest growth rate, whereas pasteurized milk showed slower increases, reflecting differences in the initial microbial load. RF pasteurization resulted in lower microbial counts and growth rates than LTLT, likely due to differences in sublethal cellular injury affecting microbial resuscitation and subsequent proliferation over time [55].

**Figure 4:**
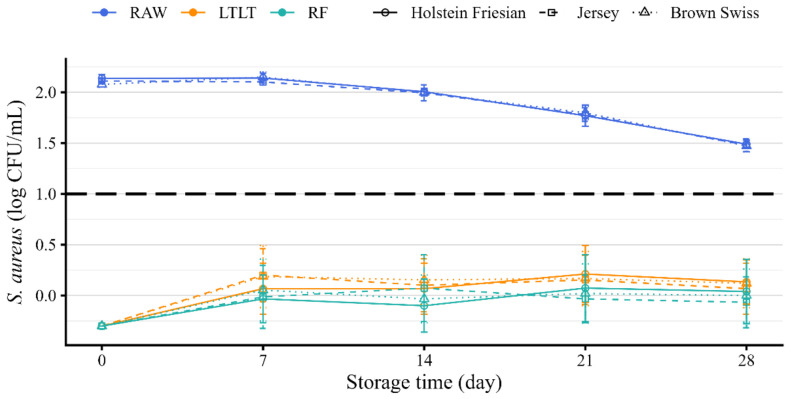
Changes in *S. aureus* of milk from Holstein Friesian, Jersey, and Brown Swiss subjected to different pasteurization methods (Raw, LTLT, and RF) during refrigerated storage (4 °C) for 28 days. The dashed horizontal line denotes the regulatory APC limit (1.00 log CFU/mL).

Pathogenic microorganisms present in raw milk, particularly those associated with mastitis resulting from inadequate farm management such as *S. aureus* were also evaluated. As shown in Figure 4, the average *S. aureus* count across all breeds in raw milk on day 0 was 2.10 log CFU/mL, whereas the organism was not detected after pasteurization. At day 28, RF-treated milk showed an *S. aureus* count of not detected, while LTLT-treated milk recorded 0.106 log CFU/mL, indicating that RF pasteurization was more effective than LTLT in controlling *S. aureus*. In raw milk, *S. aureus* counts declined during storage (days 7–28), decreasing from 2.130 log CFU/mL to not detected. This reduction is likely due to the activity of lactic acid bacteria and psychrotrophic microorganisms, which produce organic acids, bacteriocins, and other inhibitory compounds [56]. Moreover, *S. aureus* does not proliferate at low temperatures, leading to a decline in viable counts during refrigerated storage [57].

**Figure 5:**
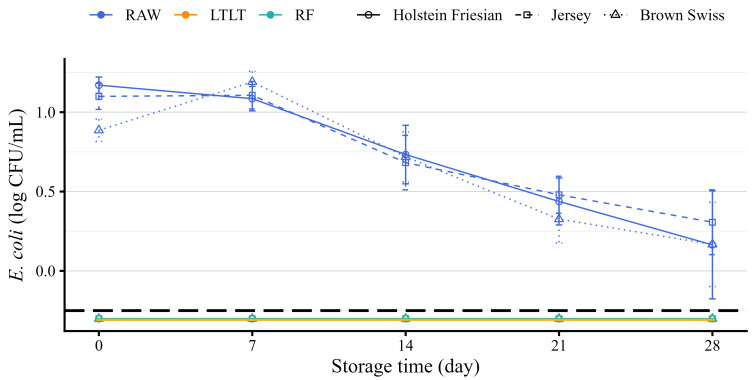
Changes in *E. coli* of milk from Holstein Friesian, Jersey, and Brown Swiss subjected to different pasteurization methods (Raw, LTLT, and RF) during refrigerated storage (4 °C) for 28 days. *E. coli* was not detected (<1 CFU/mL) in all samples throughout storage.

The enumeration of *E. coli* is presented in Figure 5. Both LTLT and RF pasteurization effectively inactivated *E. coli*, with no detectable levels after processing. As a Gram-negative bacterium, *E. coli* is highly heat sensitive; thermal treatment disrupts cell membranes and impairs osmotic regulation, resulting in cell death [58]. Similar to *S. aureus*, *E. coli* counts in raw milk decreased during storage, declining from 1.127 log CFU/mL to 0.212 log CFU/mL between days 7 and 28.

**Figure 6:**
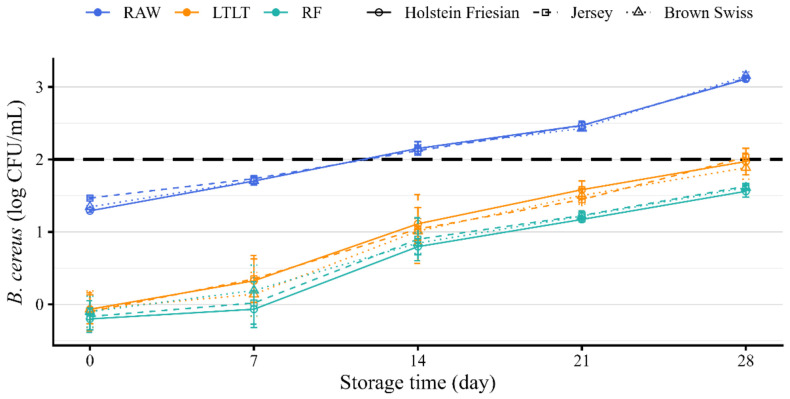
Changes in *B. cereus* in milk from Holstein Friesian, Jersey, and Brown Swiss breeds subjected to different pasteurization methods (Raw, LTLT, and RF) during refrigerated storage (4 °C) for 28 days. The dashed horizontal line denotes the regulatory APC limit (2.00 log CFU/mL).

An important pathogenic bacterium associated with raw milk and ready-to-drink dairy processing facilities is *B. cereus*. As shown in Figure 6, raw milk contained an average *B. cereus* count of 1.367 log CFU/mL across all breeds. After pasteurization, counts decreased to not detected following LTLT treatment and not detected following RF processing. During refrigerated storage (days 7–28), *B. cereus* levels in LTLT-treated milk were higher than those in RF-treated milk. This difference is attributed to the bacterium’s ability to form heat-resistant spores [59]. While pasteurization effectively destroys vegetative cells, it is insufficient to eliminate *B. cereus* spores, which can germinate during storage and lead to increased bacterial counts between days 14 and 28 [60]. Contamination with *B. cereus* is associated with foodborne illness, including diarrheal and emetic syndromes, and remains a significant concern in pasteurized milk processing facilities [59].

**Figure 7:**
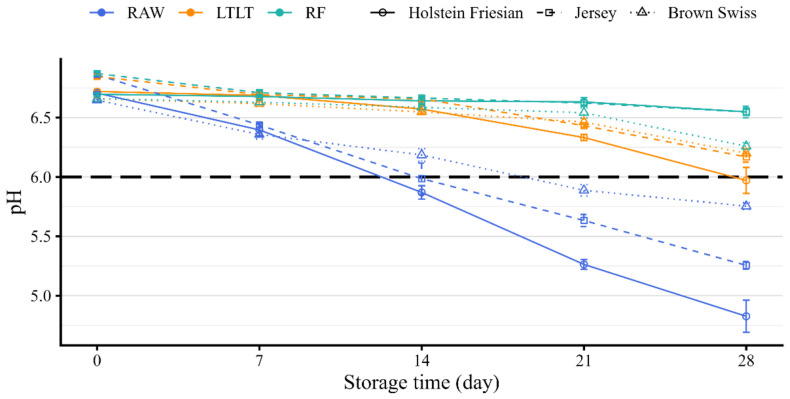
Changes in pH of milk from Holstein Friesian, Jersey, and Brown Swiss subjected to different pasteurization methods (Raw, LTLT, and RF) during refrigerated storage (4 °C) for 28 days. The dashed line denotes a reference pH value (6.0) indicating quality deterioration.

The pH level is a key indicator for monitoring the quality and shelf life of cow’s milk. As shown in Figure 7, no statistically significant differences (*p* > 0.05) were observed in pH between raw and pasteurized milk at the initial stages of storage. By day 14, raw milk exhibited a greater decline in pH compared with pasteurized milk. At day 28, the average pH across all breeds was 6.113 for LTLT-treated milk and 6.451 for RF-treated milk, indicating that RF pasteurization was more effective in maintaining pH stability than LTLT. In general, the decrease in pH during storage is associated with the activity of lactic acid bacteria (LAB) and psychrotrophic microorganisms, which produce organic acids during growth [56]. Breed-specific evaluation showed that Jersey and Brown Swiss milk, which typically contain higher fat levels than Holstein Friesian milk, exhibited smaller reductions in pH. This effect is attributed to the protective role of milk fat and the milk fat globule membrane (MFGM), which contains phospholipids and proteins with antimicrobial properties. Furthermore, higher-fat milk has greater total solids and protein content, resulting in an increased buffering capacity and requiring more acid production to achieve the same pH decrease as lower-fat milk [61].

### 3.3. Economic Feasibility Analysis

#### 3.3.1. Initial Investment

Table 12 presents the initial investment costs for the two different pasteurization processes. Straight-line depreciation was applied over a 10-year period. One working day was defined as 8 h, with 300 working days per year, resulting in 2400 operating hours annually. For the LTLT process, the pasteurization equipment has a capacity of 10 L per batch using a double-jacketed boiler with water as the heating medium, requiring 30 min per batch. This corresponds to a production capacity of 20 L per hour, or 160 L per day, equivalent to 48,000 L per year. In contrast, the RF pasteurization system achieved a production capacity of 33 L per hour, or 264 L per day, equivalent to 79,200 L per year. The listed capital investment costs excluded land, concrete flooring, roofing, and related electrical installation expenses. The results showed that the initial investment for LTLT pasteurization totaled 190.23 USD, consisting of a gas burner and a double-jacketed boiler system using water as the heat transfer medium. The annual depreciation cost was 19.02 USD. In comparison, the RF pasteurization system required an initial investment of 15,852.89 USD, with an annual depreciation cost of 1585.29 USD. Both methods were calculated based on a 10-year service life. When considering production capacity, the RF pasteurization system increased output by approximately 65% compared with the LTLT method, due to its rapid heating rate and higher throughput than the conventional process [16].

#### 3.3.2. Pasteurization Operating Costs

Table 13 presents the operating costs of the two pasteurization processes. For the LTLT system, the low-pressure gas burner was assumed to consume 0.25 kg of gas per hour, with an average gas price of 0.95 USD per kilogram, equivalent to 0.24 USD per hour. For the RF system, electricity consumption was 8 kW per hour, equivalent to 1.01 USD per hour, based on an electricity rate of 0.13 USD per kWh. Labor cost was set at 11.10 USD per person per day, and annual maintenance cost was calculated at 3% of the total investment value. The results indicated that the total annual operating cost of LTLT pasteurization was 3905.52 USD, whereas RF pasteurization totaled 6227.52 USD. Although the RF system had higher energy costs than the LTLT system, it also provided a higher production capacity. As a result, the cost per liter was 3.62% lower for RF compared with LTLT. This outcome reflects the greater efficiency in heat transfer than water-based heating, thereby reducing the overall energy consumption [62]. A 3% annual maintenance cost was assumed based on commonly applied estimates for food-processing equipment. Because the RF system contains more complex electronic components than the conventional LTLT system, additional sensitivity analyses using 5% and 7% maintenance rates were also evaluated. Under these scenarios, the RF system continued to show positive NPV and BCR values greater than 1, although the economic advantage was reduced. These results suggest that the economic feasibility of the RF system remains relatively robust to variation in maintenance-cost assumptions.

#### 3.3.3. Revenue from Pasteurization

Table 14 presents the revenue generated from the two pasteurization processes. In this analysis, the raw material cost and selling price for RF pasteurization were assumed to be the same as those for LTLT pasteurization, with raw milk priced at 0.92 USD per liter and a selling price of 1.27 USD per liter. At this selling price and raw milk cost, both processes generated a gross profit of 0.35 USD per liter. However, due to differences in production capacity, the total annual revenue varied between the two systems. LTLT pasteurization generated 16,740.65 USD per year, which was 10,849.71 USD less than RF pasteurization. This difference corresponded directly to the higher production capacity of the RF system. In addition, improved heating efficiency and reduced overall energy consumption contributed to increased income for farmers. The lower energy usage also provided an environmental benefit by reducing greenhouse gas emission and contributing to the mitigation of global warming impacts [63].

#### 3.3.4. Project Feasibility Analysis

Table 15 and Table 16 present the cash flow statements over a 10-year project period for LTLT and RF pasteurization systems. The net annual cash flow after expenses for LTLT pasteurization was 12,835.13 USD per year. With an initial investment of 190.23 USD, the payback period was less than one month. The cumulative net cash flow at year 10 reached 128,161.07 USD. In contrast, RF pasteurization required a substantially higher initial investment of 15,852.89 USD. However, the net annual cash flow after expenses was 21,382.37 USD per year, resulting in a payback period of approximately 6.8 months. The cumulative net cash flow at year 10 was 197,970.83 USD. These results indicate that RF pasteurization increased farmers’ income by 54.47% compared with conventional pasteurization. Although the initial investment cost of the two systems differed considerably, the higher operational efficiency of the RF system resulted in greater overall economic feasibility than the traditional method [64].

Table 17 presents the results of the economic analysis, including NPV, IRR, BCR and PB. Using a discount rate of 7.042%, the NPV of LTLT pasteurization was calculated at 90,004.52 USD, which was lower than that of RF pasteurization at 134,721.78 USD. This indicates that the novel RF pasteurization approach is economically viable and provides greater long-term economic value. The IRR for LTLT pasteurization was 6747%, reflecting the very low initial investment of 190.23 USD, whereas the IRR for RF pasteurization was 134%, exceeding the discount rate and confirming its economic feasibility. The extremely high IRR value calculated for the LTLT system is primarily attributable to the very low initial investment relative to the projected annual cash flow. Therefore, this value should not be interpreted as a practical discount-rate threshold in the conventional financial sense. In the present comparison, NPV and benefit–cost ratio are considered more informative indicators for evaluating economic performance between the two systems. The BCR values were 4.26 for LTLT and 3.25 for RF, demonstrating that both approaches yield benefits greater than their costs and are financially worthwhile. Regarding the payback period, LTLT recovered its investment in less than one month, while RF achieved payback within 6.8 months. Both processes therefore offer rapid capital recovery relative to the 10-year project lifespan. However, although LTLT shows an exceptionally high IRR due to its minimal initial investment, consideration of NPV and production capacity indicates that RF pasteurization provides superior overall economic performance. These findings highlight the investment value of RF technology and its ability to achieve payback within a timeframe substantially shorter than the equipment’s service life and serves as an alternative for milk pasteurization in small-scale industrial applications [44,45,65].

However, The base-case NPV assumes full-capacity operation for both systems. Because the RF system’s capacity (33 L/h) exceeds that of LTLT (20 L/h) by approximately 65%, its economic advantage depends on the market’s ability to absorb the additional production volume. Under a demand-constrained scenario in which RF output is limited to the same production volume as the LTLT system, RF processing remains economically viable, although with a reduced NPV advantage. Therefore, the base-case economic estimates should be interpreted with caution.

In addition, this novel pasteurization approach has the potential to expand the market for pasteurized cow’s milk by extending shelf life up to three times longer than conventional methods. This extended shelf life enhances the market reach of milk produced by dairy farmers in Northern Thailand and aligns with current efforts to reduce food loss. Consequently, it can contribute to increased and sustainable long-term income for farmers. However, the high initial investment cost may represent a barrier for individual farmers due to the relatively expensive equipment required. Therefore, governmental support mechanisms are essential to promote wider adoption of this innovation. Policy support could facilitate technology dissemination through dairy cooperatives and expand implementation to niche farmer groups, such as buffalo milk and goat milk producers, thereby increasing value creation across diversified dairy sectors.

## 4. Conclusions

This study demonstrates the feasibility of RF heating technology for cow milk pasteurization under prototype-scale continuous-flow processing conditions. Among the conditions evaluated, heating at 92 °C for 50 s provided the most suitable balance between microbial reduction, preservation of milk composition and physical properties, and energy utilization. Under these conditions, total microbial counts were significantly reduced, and no detectable *S. aureus*, *B. cereus*, and *E. coli* were observed.

RF pasteurization also extended the refrigerated shelf life of milk compared with the conventional process evaluated in this study. In addition, the RF process maintained relatively consistent quality characteristics across milk obtained from different dairy cow breeds, suggesting potential applicability to mixed-breed dairy systems.

From an economic perspective, the RF prototype system showed potential for improving processing efficiency and reducing energy consumption under the tested conditions. However, direct thermal-equivalent comparison with conventional pasteurization was not performed in the present study, and therefore RF-specific effects could not be conclusively distinguished from thermal effects alone.

Furthermore, vitamin retention, lipid oxidation, heating uniformity, sensory characteristics, and consumer acceptability were not assessed and remain important objectives for future research. Future studies should therefore focus on thermal-equivalent comparisons with conventional heating, scale-up evaluation, and comprehensive sensory and consumer acceptance assessment to support the potential commercial application of RF-assisted milk pasteurization.

## Figures and Tables

**Table 1:** Configuration of milk flow rate and holding time parameters.

Pump Frequency (Hz)	Flow Rate (L/h)	Holding Time (s)
15	15.91 ± 0.05	105.43 ± 0.32
16	23.74 ± 0.14	70.67 ± 0.40
17	33.35 ± 0.18	50.30 ± 0.26
18	41.43 ± 0.71	40.50 ± 0.70
19	49.88 ± 0.37	33.63 ± 0.25
20	56.17 ± 0.60	29.87 ± 0.32
21	77.80 ± 0.58	21.57 ± 0.15
22	86.47 ± 0.45	19.40 ± 0.10
23	99.08 ± 0.89	16.93 ± 0.15
24	102.29 ± 0.62	16.40 ± 0.10
25	108.48 ± 1.45	15.47 ± 0.21

Data are expressed as mean ± SD in triplicate.

**Table 2:** Evaluation of optimal processing conditions for RF pasteurization.

Treatments	Process	Temperature (°C)	Time (s)
RAW	Raw milk	−	−
LTLT	LTLT Pasteurization	63	1800
RF7220	RF Pasteurization	72	20
RF7250	RF Pasteurization	72	50
RF72100	RF Pasteurization	72	100
RF8220	RF Pasteurization	82	20
RF8250	RF Pasteurization	82	50
RF82100	RF Pasteurization	82	100
RF9220	RF Pasteurization	92	20
RF9250	RF Pasteurization	92	50
RF92100	RF Pasteurization	92	100

Note: Raw milk; milk that has not undergone any heat treatment (Temperature = –, Time = –), LTLT; Low Temperature–Long Time pasteurization (conventional pasteurization at 63 °C for 1800 s), RF; Radio Frequency pasteurization.

**Table 3:** RF pasteurization of high-quality cow’s milk produced in northern Thailand.

Treatments	Dairy Breed	Process	Temperature (°C)	Time (s)
HRAW	Holstein Friesian	Raw milk	–	–
JRAW	Jersey	Raw milk	–	–
BRAW	Brown Swiss	Raw milk	–	–
HLTLT	Holstein Friesian	LTLT Pasteurization	63	1800
JLTLT	Jersey	LTLT Pasteurization	63	1800
BLTLT	Brown Swiss	LTLT Pasteurization	63	1800
HRF	Holstein Friesian	RF Pasteurization	Most effective	
JRF	Jersey	RF Pasteurization	Most effective	
BRF	Brown Swiss	RF Pasteurization	Most effective	

Note: Raw milk; milk that has not undergone any heat treatment (Temperature = –, Time = –), LTLT; Low Temperature–Long Time pasteurization (conventional pasteurization at 63 °C for 1800 s), RF; Radio Frequency pasteurization, The most effective; optimal temperature–time condition based on milk quality and microbial inactivation.

**Table 4:** Electrical energy consumption, specific energy consumption, and specific energy costs of milk processed using RF thermal pasteurization.

Treatment	Power Consumption (kWh)	Specific Energy Consumption (kWh/L)	Energy Cost (USD/L)
RF72/20	7.03 ± 0.1155 ^a^	0.09 ± 0.0021 ^a^	0.01 ± 0.0003 ^a^
RF72/50	7.33 ± 0.1528 ^ab^	0.22 ± 0.0057 ^b^	0.03 ± 0.0007 ^b^
RF72/100	7.70 ± 0.2000 ^b^	0.48 ± 0.0123 ^d^	0.06 ± 0.0016 ^d^
RF82/20	7.33 ± 0.1528 ^ab^	0.09 ± 0.0013 ^a^	0.01 ± 0.0002 ^a^
RF82/50	7.60 ± 0.2000 ^b^	0.23 ± 0.0054 ^b^	0.03 ± 0.0007 ^b^
RF82/100	8.20 ± 0.1000 ^c^	0.53 ± 0.0077 ^e^	0.07 ± 0.0010 ^e^
RF92/20	8.27 ± 0.1528 ^c^	0.11 ± 0.0021 ^a^	0.01 ± 0.0003 ^a^
RF92/50	8.67 ± 0.1528 ^c^	0.26 ± 0.0050 ^c^	0.03 ± 0.0006 ^c^
RF92/100	9.20 ± 0.2646 ^d^	0.59 ± 0.0141 ^f^	0.07 ± 0.0018 ^f^

Data are expressed as mean ± SD (n = 3). Different superscript letters (^a^–^f^) within the same column indicate significant differences (*p* < 0.05).

**Table 5:** Chemical composition, physical quality parameters, and somatic cell counts of milk following RF pasteurization.

Treatment	Milk Quality							
	Fat, %	Protein, %	Lactose, %	TS, %	SNF, %	SCC	FP (°C)	pH
RF72/20	3.36 ± 0.31	3.28 ± 0.01	4.44 ± 0.03 ^ab^	12.10 ± 0.26	8.74 ± 0.05 ^bc^	205.33 ± 25.01	0.527 ± 0.00	6.79 ± 0.01 ^abc^
RF72/50	3.48 ± 0.18	3.24 ± 0.05	4.41 ± 0.03 ^b^	12.19 ± 0.14	8.71 ± 0.04 ^c^	198.33 ± 18.93	0.526 ± 0.00	6.78 ± 0.01 ^bc^
RF72/100	3.53 ± 0.04	3.27 ± 0.02	4.42 ± 0.00 ^b^	12.23 ± 0.04	8.70 ± 0.00 ^c^	209.33 ± 10.02	0.526 ± 0.00	6.78 ± 0.01 ^bc^
RF82/20	3.11 ± 0.51	3.25 ± 0.03	4.47 ± 0.05 ^ab^	11.95 ± 0.41	8.84 ± 0.11 ^abc^	211.00 ± 8.54	0.527 ± 0.00	6.80 ± 0.01 ^ab^
RF82/50	3.40 ± 0.09	3.24 ± 0.01	4.47 ± 0.01 ^a^	12.21 ± 0.06	8.81 ± 0.03 ^abc^	192.00 ± 11.27	0.529 ± 0.00	6.81 ± 0.01 ^a^
RF82/100	2.99 ± 0.37	3.27 ± 0.02	4.51 ± 0.03 ^a^	11.88 ± 0.31	8.90 ± 0.06 ^a^	206.67 ± 20.82	0.528 ± 0.00	6.78 ± 0.00 ^bc^
RF92/20	3.19 ± 0.23	3.27 ± 0.02	4.49 ± 0.01 ^a^	12.07 ± 0.19	8.88 ± 0.04 ^ab^	205.33 ± 21.73	0.531 ± 0.00	6.77 ± 0.03 ^c^
RF92/50	3.27 ± 0.19	3.27 ± 0.02	4.49 ± 0.02 ^a^	12.14 ± 0.16	8.87 ± 0.04 ^ab^	215.67 ± 16.04	0.528 ± 0.00	6.78 ± 0.00 ^bc^
RF92/100	3.03 ± 0.12	3.28 ± 0.01	4.51 ± 0.01 ^a^	11.95 ± 0.10	8.92 ± 0.02 ^a^	210.00 ± 21.00	0.529 ± 0.00	6.79 ± 0.01 ^abc^

Data are expressed as mean ± SD (n = 3). Different superscript letters (^a^–^c^) within the same column indicate significant differences (*p* < 0.05). TS = Total solid; SNF = Solid non fat; SCC = Somatic Cell Count (×1000 cell/mL); FP = Freezing point; RF = Radio Frequency pasteurization (Temperature (°C)/Time (s)).

**Table 6:** Microbial counts in pasteurized milk under different temperature and time conditions.

Treatment	Type of Microorganisms (log CFU/mL)						
	APC	Log Reduction	*S. aureus*	Log Reduction	*B. cereus*	Log Reduction	*E. coli*
RF72/20	2.38 ± 0.02 ^e^	3.42	1.64 ± 0.06	1.55	1.34 ± 0.13 ^c^	0.47	ND
RF72/50	2.18 ± 0.01 ^e^	3.62	ND	3.29	1.23 ± 0.08 ^c^	0.58	ND
RF72/100	1.93 ± 0.02 ^d^	3.87	ND	3.49	0.83 ± 0.16 ^b^	0.98	ND
RF82/20	1.70 ± 0.02 ^cd^	4.1	ND	3.29	0.65 ± 0.16 ^ab^	1.16	ND
RF82/50	1.59 ± 0.01 ^c^	4.21	ND	3.49	0.46 ± 0.15 ^a^	1.35	ND
RF82/100	1.48 ± 0.05 ^c^	4.32	ND	3.49	ND	1.81	ND
RF92/20	1.16 ± 0.20 ^b^	4.64	ND	3.49	ND	2.01	ND
RF92/50	0.69 ± 0.09 ^a^	5.11	ND	3.49	ND	2.11	ND
RF92/100	0.69 ± 0.09 ^a^	5.11	ND	3.49	ND	2.11	ND

Data are expressed as mean ± SD (n = 3). Different superscript letters (^a^–^e^) within the same column indicate significant differences (*p* < 0.05). Log reduction was calculated as the difference between initial microbial counts (raw milk) and treated samples. ND (<1.0 log CFU/mL). APC = Aerobic Plate Count; RF = Radio Frequency pasteurization (Temperature (°C)/Time (s)).

**Table 7:** Colour parameters of pasteurized milk under different temperature and time conditions.

Treatment	L*	a*	b*	Whiteness Index	∆E*
RF72/20	89.85 ± 0.52 ^a^	0.45 ± 0.17 ^a^	14.46 ± 0.30 ^d^	82.32 ± 0.35	1.01 ± 0.43
RF72/50	89.62 ± 0.61 ^ab^	0.41 ± 0.05 ^a^	15.30 ± 0.19 ^abc^	81.50 ± 0.19	1.66 ± 0.16
RF72/100	89.49 ± 0.16 ^abc^	0.32 ± 0.01 ^a^	15.54 ± 0.28 ^ab^	81.24 ± 0.30	1.87 ± 0.27
RF82/20	89.30 ± 0.54 ^abc^	0.12 ± 0.01 ^b^	14.85 ± 0.52 ^bcd^	81.69 ± 0.62	1.73 ± 0.50
RF82/50	88.26 ± 0.78 ^c^	0.02 ± 0.01 ^b^	14.58 ± 0.16 ^cd^	81.27 ± 0.55	2.53 ± 0.72
RF82/100	88.55 ± 0.20 ^abc^	0.05 ± 0.01 ^b^	14.62 ± 0.10 ^cd^	81.43 ± 0.20	2.25 ± 0.20
RF92/20	88.62 ± 0.27 ^abc^	−0.01 ± 0.02 ^b^	15.44 ± 0.16 ^ab^	80.82 ± 0.29	2.52 ± 0.29
RF92/50	88.32 ± 0.52 ^bc^	0.02 ± 0.01 ^b^	15.64 ± 0.15 ^a^	80.48 ± 0.40	2.84 ± 0.46
RF92/100	88.49 ± 0.25 ^bc^	0.10 ± 0.02 ^b^	15.44 ± 0.16 ^ab^	80.75 ± 0.23	2.58 ± 0.25

Data are expressed as mean ± SD (n = 3). Different superscript letters (^a^–^d^) within the same column indicate significant differences (*p* < 0.05). L* represents lightness (0 = black, 100 = white); a* represents the red (+) to green (−) coordinate; b* represents the yellow (+) to blue (−) coordinate. ∆E* indicates the total color difference compared to the control sample. Whiteness Index (WI) was calculated based on L*, a*, and b* values. RF = Radio Frequency pasteurization (Temperature (°C)/Time (s)).

**Table 8:** Comparison of milk quality between conventional LTLT pasteurization and the optimal RF treatment condition (92 °C/50 s).

Treatment	RAW	LTLT	RF92/50
Fat, %	3.40 ± 0.09	3.29 ± 0.05	3.27 ± 0.19
Protein, %	3.30 ± 0.01 ^a^	3.34 ± 0.01 ^b^	3.27 ± 0.02 ^a^
Lactose, %	4.49 ± 0.02 ^a^	4.59 ± 0.01 ^b^	4.49 ± 0.02 ^a^
TS, %	12.23 ± 0.01	12.23 ± 0.04	12.14 ± 0.16
SNF, %	8.90 ± 0.06 ^a^	9.08 ± 0.01 ^b^	8.87 ± 0.04 ^a^
SCC	210.00 ± 26.46	212.67 ± 11.93	215.67 ± 16.04
FP (°C)	0.527 ± 0.00 ^a^	0.566 ± 0.00 ^b^	0.528 ± 0.00 ^a^
pH	6.82 ± 0.01 ^a^	6.77 ± 0.03 ^b^	6.78 ± 0.00 ^ab^
APC	5.80 ± 0.04 ^b^	0.68 ± 0.24 ^a^	0.69 ± 0.09 ^a^
Log reduction	NA	5.12	5.11
*S. aureus*	3.19 ± 0.02	ND	ND
Log reduction	NA	3.49	3.49
*B. cereus*	1.81 ± 0.02	0.68 ± 0.24	ND
Log reduction	NA	1.13	2.11
*E. coli*	3.18 ± 0.06	ND	ND
Log reduction	NA	3.48	3.48
L*	90.53 ± 0.06 ^a^	89.21 ± 0.14 ^b^	88.32 ± 0.52 ^c^
a*	1.01 ± 0.03 ^a^	0.74 ± 0.02 ^b^	0.02 ± 0.01 ^c^
b*	14.16 ± 0.04 ^b^	14.45 ± 0.20 ^b^	15.64 ± 0.15 ^a^
Whiteness Index	82.94 ± 0.01 ^a^	81.95 ± 0.10 ^b^	80.48 ± 0.40 ^c^
∆E*	0.00	1.40 ± 0.10 ^a^	2.84 ± 0.46 ^b^

Data are expressed as mean ± SD (n = 3). Different superscript letters (^a^–^c^) within the same row indicate significant differences (*p* < 0.05). TS = Total solid; SNF = Solid non fat; SCC = Somatic Cell Count (×1000 cell/mL); FP = Freezing point; RF = Radio Frequency pasteurization (Temperature (°C)/Time (s)). Log reduction was calculated as the difference between initial microbial counts (raw milk) and treated samples. NA = not applicable, ND = not detected (below the detection limit, assumed as −0.30 log CFU/mL). APC = Aerobic Plate Count; RF = Radio Frequency pasteurization (Temperature (°C)/Time (s)). L* represents lightness (0 = black, 100 = white); a* represents the red (+) to green (−) coordinate; b* represents the yellow (+) to blue (−) coordinate. ∆E* indicates the total color difference compared to the control sample (RAW was used as the ΔE* reference; therefore, ΔE* = 0.00). Whiteness Index (WI) was calculated based on L*, a*, and b* values. RF = Radio Frequency pasteurization (Temperature (°C)/Time (s)).

**Table 9:** Aflatoxin M1.

Treatment	N	Max. Conc. (ng/kg)	Mean ± SD (ng/kg)
RAW	5	16.20	13.18 ± 2.11 ^b^
LTLT	3	10.00	9.83 ± 0.29 ^a^
RF72/20	3	10.20	9.23 ± 1.12 ^ab^
RF72/50	3	9.50	7.57 ± 2.61 ^a^
RF72/100	3	9.80	8.07 ± 1.50 ^ab^
RF82/20	3	8.30	7.50 ± 0.98 ^a^
RF82/50	3	9.50	7.30 ± 1.97 ^a^
RF82/100	3	8.70	8.57 ± 0.12 ^ab^
RF92/20	3	10.00	7.93 ± 3.32 ^ab^
RF92/50	3	11.20	9.00 ± 2.84 ^ab^
RF92/100	3	9.80	7.23 ± 2.23 ^a^

Data are expressed as mean ± SD. N represents the number of samples analyzed for each treatment. Different superscript letters (^a^, ^b^) within the same column indicate significant differences (*p* < 0.05). Max. Conc. = Maximum concentration.

**Table 10:** Chemical composition, physical quality parameters, and somatic cell count of milk from different dairy breeds.

Variable	Breed	RAW	LTLT	RF	*p* (Breed)	*p* (Method)	*p* (Interaction)
Fat, %	Holstein	4.557 ± 0.015 ^a^	4.557 ± 0.015 ^b^	4.557 ± 0.015 ^b^	<0.001	<0.001	<0.001
	Jersey	4.829 ± 0.015 ^a^	4.829 ± 0.015 ^b^	4.829 ± 0.015 ^c^			
	Brown Swiss	4.407 ± 0.015 ^a^	4.407 ± 0.015 ^a^	4.407 ± 0.015 ^b^			
Protein, %	Holstein	3.506 ± 0.008 ^a^	3.519 ± 0.008 ^a^	3.460 ± 0.008 ^a^	<0.001	<0.001	<0.001
	Jersey	3.762 ± 0.008 ^b^	3.788 ± 0.008 ^b^	3.617 ± 0.008 ^b^			
	Brown Swiss	3.830 ± 0.008	3.844 ± 0.008	3.826 ± 0.008			
Lactose, %	Holstein	4.148 ± 0.008 ^a^	4.221 ± 0.008 ^a^	4.120 ± 0.008 ^b^	<0.001	<0.001	<0.001
	Jersey	4.058 ± 0.008 ^b^	4.149 ± 0.008 ^a^	4.014 ± 0.008 ^c^			
	Brown Swiss	4.224 ± 0.008 ^a^	4.248 ± 0.008 ^a^	4.162 ± 0.008 ^b^			
TS, %	Holstein	13.397 ± 0.029 ^a^	12.916 ± 0.029 ^b^	11.323 ± 0.029 ^c^	<0.001	<0.001	<0.001
	Jersey	14.017 ± 0.029 ^a^	13.881 ± 0.029 ^b^	13.393 ± 0.029 ^c^			
	Brown Swiss	13.543 ± 0.029 ^a^	13.517 ± 0.029 ^a^	12.952 ± 0.029 ^b^			
SNF, %	Holstein	8.847 ± 0.020 ^b^	8.936 ± 0.020 ^a^	8.568 ± 0.020 ^c^	<0.001	<0.001	0.001
	Jersey	9.166 ± 0.020 ^a^	9.204 ± 0.020 ^a^	8.780 ± 0.020 ^b^			
	Brown Swiss	9.104 ± 0.020 ^a^	9.109 ± 0.020 ^a^	8.817 ± 0.020 ^b^			
SCC	Holstein	103.444 ± 10.071 ^a^	72.444 ± 10.071 ^b^	64.111 ± 10.071 ^b^	0.058 (ns)	<0.001	0.001
	Jersey	77.111 ± 10.071	72.667 ± 10.071	69.556 ± 10.071			
	Brown Swiss	182.333 ± 10.071 ^a^	64.889 ± 10.071 ^b^	58.111 ± 10.071 ^b^			
FP (°C)	Holstein	0.529 ± 0.006	0.525 ± 0.006	0.513 ± 0.006	0.024	<0.001	0.766 (ns)
	Jersey	0.546 ± 0.006 ^a^	0.538 ± 0.006 ^a^	0.515 ± 0.006 ^b^			
	Brown Swiss	0.526 ± 0.006	0.525 ± 0.006	0.507 ± 0.006			
pH	Holstein	6.707 ± 0.005 ^ab^	6.720 ± 0.005 ^a^	6.697 ± 0.005 ^b^	<0.001	0.366 (ns)	<0.001
	Jersey	6.860 ± 0.005 ^ab^	6.847 ± 0.005 ^b^	6.871 ± 0.005 ^a^			
	Brown Swiss	6.647 ± 0.005	6.658 ± 0.005	6.660 ± 0.005			

Data are expressed as least squares means ± SEM. Different superscript letters within the same row indicate significant differences among processing methods based on LSD pairwise comparison (*p* < 0.05). ns = not significant (*p* > 0.05); TS = total solids; SNF = solids not fat; SCC = somatic cell count (×1000 cells/mL); FP = freezing point. Raw milk = milk without heat treatment; LTLT = low-temperature long-time pasteurization at 63 °C for 30 min; RF = radio-frequency pasteurization.

**Table 11:** Colour characteristics of milk products.

Variable	Breed	RAW	LTLT	RF	*p* (Breed)	*p* (Method)	*p* (Interaction)
L*	Holstein	87.763 ± 0.143 ^c^	88.216 ± 0.143 ^b^	88.697 ± 0.143 ^a^	<0.001	<0.001	0.993 (ns)
	Jersey	86.673 ± 0.143 ^c^	87.170 ± 0.143 ^b^	87.678 ± 0.143 ^a^			
	Brown Swiss	87.252 ± 0.143 ^c^	87.671 ± 0.143 ^b^	88.269 ± 0.143 ^a^			
a*	Holstein	0.701 ± 0.019 ^a^	0.488 ± 0.019 ^b^	0.183 ± 0.019 ^c^	<0.001	<0.001	0.600 (ns)
	Jersey	1.499 ± 0.019 ^a^	1.288 ± 0.019 ^b^	0.979 ± 0.019 ^c^			
	Brown Swiss	1.087 ± 0.019 ^a^	0.930 ± 0.019 ^b^	0.596 ± 0.019 ^c^			
b*	Holstein	13.557 ± 0.067 ^c^	13.881 ± 0.067 ^b^	14.444 ± 0.067 ^a^	<0.001	<0.001	0.400 (ns)
	Jersey	16.002 ± 0.067 ^c^	16.471 ± 0.067 ^b^	17.136 ± 0.067 ^a^			
	Brown Swiss	14.426 ± 0.067 ^c^	14.884 ± 0.067 ^b^	15.402 ± 0.067 ^a^			
Whiteness Index	Holstein	81.721 ± 0.113	81.783 ± 0.113	81.655 ± 0.113	<0.001	0.286 (ns)	0.868 (ns)
	Jersey	79.119 ± 0.113	79.080 ± 0.113	78.869 ± 0.113			
	Brown Swiss	80.715 ± 0.113	80.647 ± 0.113	80.627 ± 0.113			
∆E*	Holstein	0.147 ± 0.027	0.620 ± 0.027	1.399 ± 0.027	0.059 (ns)	<0.001	0.407 (ns)
	Jersey	0.175 ± 0.027	0.738 ± 0.027	1.609 ± 0.027			
	Brown Swiss	0.137 ± 0.027	0.658 ± 0.027	1.507 ± 0.027			

Data are expressed as least squares means ± SEM. Different superscript letters within the same row indicate significant differences among processing methods based on a LSD pairwise comparison (*p* < 0.05). ns = not significant (*p* > 0.05); L* represents lightness (0 = black, 100 = white); a* represents the red (+) to green (−) coordinate; b* represents the yellow (+) to blue (−) coordinate. ∆E* indicates the total color difference compared to the control sample. Whiteness Index (WI) was calculated based on L*, a*, and b* values. Raw milk = milk that has not undergone any heat treatment (Temperature = –, Time = –); LTLT = Low Temperature–Long Time pasteurization (conventional pasteurization at 63 °C for 30 min); RF = Radio Frequency pasteurization.

**Table 12:** Initial investment costs for pasteurization systems.

Item	Method	
	LTLT Pasteurization	RF Pasteurization
Description	Stove and Double-Jacketed Boiler	RF Pasteurizer
Method Cost (USD)	126.82	15,535.83
Equipment Cost (USD)	63.41	317.06
Total (USD)	190.23	15,852.89
Depreciation (USD/year)	19.02	1585.29

Note: Depreciation was calculated using a straight-line method based on the total investment cost.

**Table 13:** Annual pasteurization operating costs.

Item	Method	
	LTLT Pasteurization	RF Pasteurization
Gas/Electricity Cost (USD)	570.70	2422.83
Labor Cost (USD)	3329.11	3329.11
Maintenance Cost (USD)	5.71	475.59
Total (USD)	3905.52	6227.52
Cost (USD/Liter)	0.08	0.08

Note: Production cost per liter was calculated based on the total operating cost divided by the annual milk processing capacity.

**Table 14:** Revenue from pasteurization processes.

Item	Method	
	LTLT Pasteurization	RF Pasteurization
Production Volume (Liters/Year)	1521.88	2511.10
Profit (USD/Liter)	0.35	0.35
Annual Profit (USD)	16,740.65	27,622.07

Note: Annual profit was estimated from the annual production volume multiplied by the profit per liter.

**Table 15:** Cash flow for LTLT pasteurization.

Year	Initial Investment	Operating Cost	Total Cost	Revenue	Net Cash Flow	Cumulative Net Cash Flow
0	190.23	0.00	190.23	0.00	−190.23	−190.23
1	0.00	3905.52	3905.52	16,740.65	12,835.13	12,644.90
2	0.00	3905.52	3905.52	16,740.65	12,835.13	25,480.03
3	0.00	3905.52	3905.52	16,740.65	12,835.13	38,315.16
4	0.00	3905.52	3905.52	16,740.65	12,835.13	51,150.29
5	0.00	3905.52	3905.52	16,740.65	12,835.13	63,985.42
6	0.00	3905.52	3905.52	16,740.65	12,835.13	76,820.55
7	0.00	3905.52	3905.52	16,740.65	12,835.13	89,655.68
8	0.00	3905.52	3905.52	16,740.65	12,835.13	102,490.81
9	0.00	3905.52	3905.52	16,740.65	12,835.13	115,325.94
10	0.00	3905.52	3905.52	16,740.65	12,835.13	128,161.07

Note: Net cash flow was calculated as revenue minus total cost, while cumulative net cash flow represents the accumulated value over the project lifetime.

**Table 16:** Cash flow for RF pasteurization.

Year	Initial Investment	Operating Cost	Total Cost	Revenue	Net Cash Flow	Cumulative Net Cash Flow
0	15,852.89	0.00	15,852.89	0.00	−15,852.89	−15,852.89
1	0.00	6239.70	6239.70	27,622.07	21,382.37	5529.49
2	0.00	6239.70	6239.70	27,622.07	21,382.37	26,911.86
3	0.00	6239.70	6239.70	27,622.07	21,382.37	48,294.23
4	0.00	6239.70	6239.70	27,622.07	21,382.37	69,676.60
5	0.00	6239.70	6239.70	27,622.07	21,382.37	91,058.97
6	0.00	6239.70	6239.70	27,622.07	21,382.37	112,441.34
7	0.00	6239.70	6239.70	27,622.07	21,382.37	133,823.72
8	0.00	6239.70	6239.70	27,622.07	21,382.37	155,206.09
9	0.00	6239.70	6239.70	27,622.07	21,382.37	176,588.46
10	0.00	6239.70	6239.70	27,622.07	21,382.37	197,970.83

Note: Net cash flow was calculated as revenue minus total cost, while cumulative net cash flow represents the accumulated financial return throughout the project lifetime.

**Table 17:** Economic analysis of pasteurization systems.

Method	NPV	IRR	BCR	PB
LTLT Pasteurization	90,004.52	6747%	4.26	<1 months
RF Pasteurization	134,721.78	134%	3.25	6.8 months

Note: NPV, IRR, BCR, and payback period were calculated based on a 10-year project lifetime using the projected cash flow data.

## Data Availability

The data presented in this study are available from the corresponding author upon reasonable request.
